# Vitamin E but Not GSH Decreases Reactive Oxygen Species Accumulation and Enhances Sperm Production during In Vitro Maturation of Frozen-Thawed Prepubertal Mouse Testicular Tissue

**DOI:** 10.3390/ijms20215380

**Published:** 2019-10-29

**Authors:** Brahim Arkoun, Ludovic Galas, Ludovic Dumont, Aurélie Rives, Justine Saulnier, Marion Delessard, Christine Rondanino, Nathalie Rives

**Affiliations:** 1 Normandie Univ, UNIROUEN, EA 4308 “Gametogenesis and Gamete Quality”, Rouen University Hospital, Biology of Reproduction–CECOS laboratory, 76000 Rouen, France; brahim.arkoun@gmail.com (B.A.); ludovic.dumont1@univ-rouen.fr (L.D.); aurelie.rives@chu-rouen.fr (A.R.); justine.saulnier@univ-rouen.fr (J.S.); marion.delessard@univ-rouen.fr (M.D.); christine.rondanino@univ-rouen.fr (C.R.); 2 Normandie Univ, UNIROUEN, INSERM, PRIMACEN, 76000 Rouen, France; ludovic.galas@univ-rouen.fr

**Keywords:** cryopreservation, in vitro spermatogenesis, prepubertal testicular tissue, reactive oxygen species, reduced glutathione, vitamin E

## Abstract

Freezing–thawing procedures and in vitro culture conditions are considered as a source of stress associated with increased reactive oxygen species (ROS) generation, leading to a damaged cell aerobic metabolism and consequently to oxidative stress. In the present study, we sought to investigate whether vitamin E (Vit E) or reduced glutathione (GSH) enhances sperm production by decreasing ROS accumulation during in vitro maturation of prepubertal mice testes. Testes of prepubertal mice were cryopreserved using a freezing medium supplemented or not supplemented with Vit E and were cultured after thawing. In presence of Rol alone in culture medium, frozen-thawed (F-T) testicular tissues exhibited a higher ROS accumulation than fresh tissue during in vitro culture. However, Vit E supplementation in freezing, thawing, and culture media significantly decreased cytoplasmic ROS accumulation in F-T testicular tissue during in vitro maturation when compared with F-T testicular tissue cultured in the presence of Rol alone, whereas GSH supplementation in culture medium significantly increased ROS accumulation associated with cytolysis and tissue disintegration. Vit E but not GSH promoted a better in vitro sperm production and was a suitable ROS scavenger and effective molecule to improve the yield of in vitro spermatogenesis from F-T prepubertal mice testes. The prevention of oxidative stress in the cytoplasmic compartment should be regarded as a potential strategy for improving testicular tissue viability and functionality during the freeze–thaw procedure and in vitro maturation.

## 1. Introduction

Testicular tissue cryopreservation is a prerequisite and the only potential strategy for fertility preservation in prepubertal boys affected by cancer [1]. Controlled slow freezing is considered as the conventional procedure [1,2,3], whereas vitrification has been proposed as a promising technique for testicular tissue banking [2,4,5]. Currently, the choice of the most efficient protocol is still lacking [3]. Despite the use of cryoprotectants and optimized protocols that seem to maintain post-thaw structural and functional integrity of testicular tissue, several other parameters should be improved, notably concerning the deleterious effects of freezing and thawing steps [3]. These chemical and physical conditions could damage cell aerobic metabolism and consequently induce oxidative stress associated with high levels of reactive oxygen species (ROS) generation [6].

ROS comprise both free radical and non-free radical oxygen-derived reactive molecules that are constantly generated, as part of the normal aerobic life, during the intermediate steps of oxygen reduction along the mitochondrial electron transport chain [6]. Intracellular ROS include superoxide anion radical (O_2_^•−^), hydroxyl radical (^•^OH), and hydrogen peroxide (H_2_O_2_). Under normal physiological circumstances, ROS may be beneficial or even indispensable in the complex process of male germ cell proliferation and maturation [7]. ROS are involved in both the proliferation and differentiation of different stem cells including spermatogonial stem cells (SSCs) and play a critical and positive role on self-renewal of SSCs [8].

However, when the proliferation/differentiation balance fails, an excessive ROS generation leads to an accumulation of these molecules in the tissue and decreases the chance of cell survival, modifying susceptible molecules including DNA, lipids, and proteins [9,10,11]. Several studies previously reported that the cryopreservation procedure is associated with ROS level variations in mammalian reproductive cells. Indeed, freeze–thaw process is considered as an extreme stressor that can modify the structure and integrity of the cell, such as spermatozoa plasma membrane [12]. Freeze–thaw procedures increase DNA oxidative damage and fragmentation levels, causing post-thaw spermatozoa to have decreased motility and viability [13]. In addition, it was demonstrated that cold shock during sperm cryopreservation is associated with oxidative stress and ROS generation [14]. In ovarian tissue, cryopreservation increased ROS levels after warming [15,16], which was associated with a high number of apoptotic cells [15].

Moreover, the use of in vitro culture techniques come with their own challenges because the in vitro micro-environment is not as ideal as the in vivo micro-environment, where ROS homeostasis is better maintained by the natural antioxidants system. For example, the composition of culture media used for human oocytes and pre-implantation embryos has a direct influence on the rate of ROS formation that impacts embryo quality and subsequently fertilization success [17]. At the tissue level, our group demonstrated the beneficial effect of retinol (Rol, vitamin A) in improving the in vitro differentiation of SSCs into spermatozoa from fresh, slow frozen and vitrified prepubertal mice testicular tissues after 30 days (D) of culture using the agarose gel system [18,19,20]. However, even though Rol or follicle stimulating hormone (FSH) and luteinizing hormone (LH) influenced the course of in vitro spermatogenesis, the yield of this process was decreased during long-term culture, notably at D60 [18]. In addition, it has been previously demonstrated that in vitro production of spermatozoa from fresh prepubertal mice testicular tissues decreased significantly during long-term culture from D45 to D60 [21]. However, a long-term ex vivo maintenance of mice testicular tissue can be improved using a microfluidic device that may reduce the toxicity of direct exposure to oxygen [22,23].

In vitro spermatogenesis failure is characterized by a decreased cell survival with germ cell loss that could be associated with excessive ROS generation during culture. Recently, our team reported complete achievement of in vitro spermatogenesis with the generation of spermatozoa from prepubertal mouse testicular tissue previously cryopreserved using an optimized controlled slow freezing protocol [24]. However, spermatogenesis efficiency, in terms of in vitro sperm production, was highly reduced with frozen–thawed (F-T) testicular tissue when compared with the fresh tissue control at D30 of culture [24,25]. Taken together, the freezing and thawing procedures followed by an in vitro culture could potentially influence ROS accumulation in testicular tissue, increasing the risk of cell or tissue damage and, thus, compromising the course of in vitro spermatogenesis.

To counteract the harmful effects of ROS, many studies developed antioxidant strategies, among which vitamin E (Vit E) or reduced glutathione (GSH) were reported to improve post-thaw semen parameters. Vit E is a well-established naturally-occurring liposoluble antioxidant and its most active form, α-tocopherol, quenches hydrogen peroxide, superoxide anion, and hydroxyl anions, and breaks peroxidation chain reactions by scavenging peroxyl radicals in lipids of the plasma membrane [26]. Addition of Vit E to the cryomedium enhanced post-thaw motility of human spermatozoa [27,28,29]. Furthermore, Vit E supplementation in the cryopreservation medium reduced the lipid peroxidation potential and improved semen quality of F-T bovine sperm [30]. Excessive levels of ROS attack the sperm membrane by the oxidation of unsaturated fatty acids, which decreases the fluidity and increases the membrane permeability [31]. Testicular tissue also contains large quantities of highly unsaturated fatty acids that could render it very vulnerable to ROS attack, notably during freezing and thawing steps [11]. Cryopreservation of isolated prepubertal mouse SSC in the presence of catalase and α-tocopherol reduced ROS production and apoptosis [32], increasing SSC viability and the ability to differentiate into spermatozoa after transplantation into seminiferous tubules [33]. GSH is ubiquitously distributed in live cells and plays an important role in the intracellular defense mechanism against oxidative stress. Glutathione peroxidase catalyzes the reduction of hydrogen peroxide into H_2_O and lipoperoxides into alkyl alcohols via GSH. The freezing procedure is known to induce a decrease in GSH content in human [34] and domestic animal spermatozoa [35,36,37]. The addition of GSH to the thawing extender resulted in a higher motility in goat spermatozoa associated with higher viability with intact acrosome, a reduction in ROS generation, and a lower chromatin condensation [38].

To the best of our knowledge, only one study has evaluated oxidative stress in F-T prepubertal testicular fragment from bovine calf [39]. However, no study has evaluated ROS accumulation in F-T prepubertal testicular tissue during long-term in vitro culture and its impact on the ability of the immature testicular tissue to generate spermatozoa. In the current study, the effects of the supplementation of cryopreservation, thawing, and culture media with Vit E or GSH on ROS accumulation were explored in live F-T testicular tissue during in vitro culture using an original protocol by ex vivo imaging through confocal macroscopy. In addition, in vitro sperm production from F-T prepubertal mouse testicular tissues was investigated.

## 2. Results

### 2.1. Detection of ROS in Cell Suspension and in Testicular Explants

#### 2.1.1. Detection in Testicular Cell Suspension

**Figure 1:**
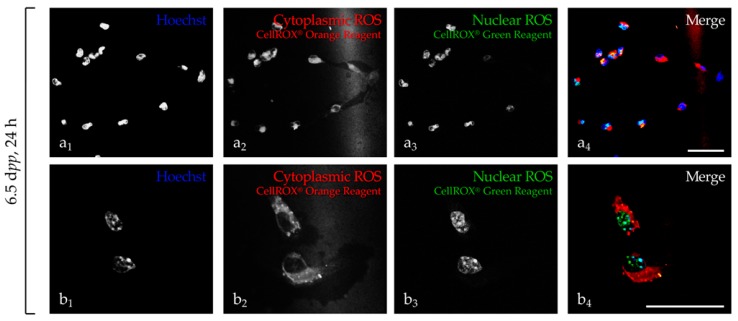
Evaluation of reactive oxygen species (ROS) probes localization in 6.5 dpp mice testicular cell suspension. (**a**,**b**) ROS signal localization after 24 h of in vitro culture of testicular cells. Testicular cells, previously incubated (30 min) with ROS probes exhibited an orange fluorescence signal present in the cytoplasm (**a_2_**,**b_2_**), while the green signal (**a_3_**,**b_3_**) was superposed (**a_4_**,**b_4_**) to Hoechst nucleus staining (**a_1_**,**b_1_**) confirming their cellular specific localization. To visualize ROS generation in testicular cells, the preparation was illuminated with a 488 nm (green) or 532 nm (orange) wavelength light. The green fluorescence emission was detected from 500–530 nm and the orange fluorescence emission from 550–580 nm. Photomicrographs were acquired with a ×63 objective and optical zoom (×1 for (**a_1_**–**a_4_**); ×2 for (**b_1_**–**b_4_**)) was adjusted to obtain a large view (**a_1_**–**a_4_**) or high magnification (**b_1_**–**b_4_**) of labeled testicular cells. The scale bar represents 40 µm. dpp, days post-partum; h, hour; H_2_O_2_, hydrogen peroxide; ROS, reactive oxygen species.

The signal emitted by the CellROX orange probe was specifically localized into the cytoplasm of the cells named “cytoplasmic” ROS in the current study (Figure 1a_2_,b_2_). In contrast, signals emitted by the CellROX green probe (Figure 1a_3_,b_3_) and Hoechst (Figure 1a_1_,b_1_) overlay with the nucleus named “nuclear” ROS in the current study (Figure 1a_4_,b_4_).

#### 2.1.2. Detection in Testicular Explants

**Figure 2:**
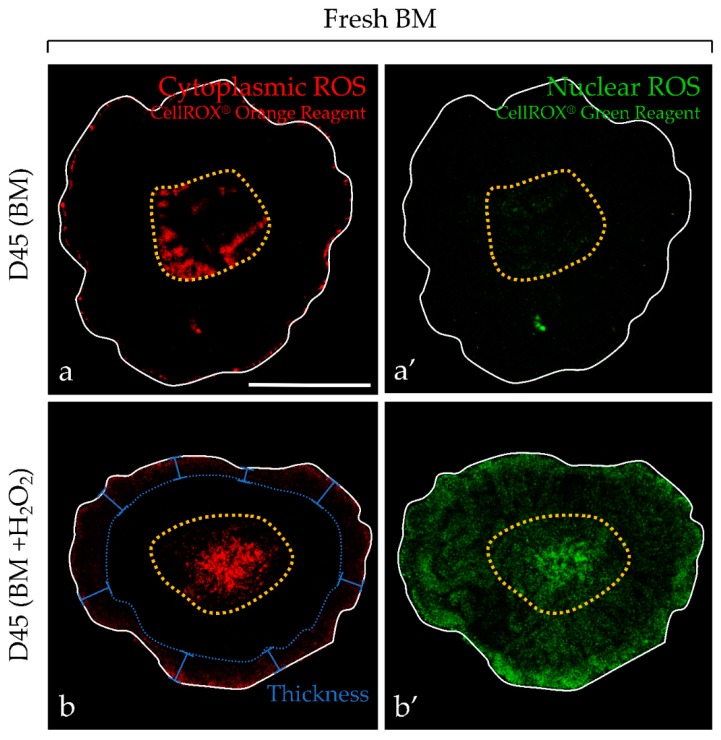
ROS detection in frozen-thawed and living testicular explants using the CellROX kit probes at D45 of culture using ex vivo imaging by confocal macroscopy. To control the specificity of the signal, frozen-thawed (F-T) testicular explants were cultured with basal medium (BM) (**a**,**a’**) or incubated with BM supplemented with 200 µM H_2_O_2_ (**b**,**b’**) overnight as positive controls. Fluorescent signal intensity appeared stronger with H_2_O_2_, either for CellROX orange reagent or green reagent, when compared with BM control. The solid line delineates the outline of the testicular fragment. Necrotic central area is delimited with yellow dotted circle and was not taken into consideration in the peripheral ROS-positive thickness region evaluation (dotted blue line). Photomicrographs were captured with a 1.1 optical zoom at a ×4.9 total magnification. The scale bar represents 1 mm. BM, basal medium; D, day; H_2_O_2_, hydrogen peroxide; ROS, reactive oxygen species.

**Figure 3:**
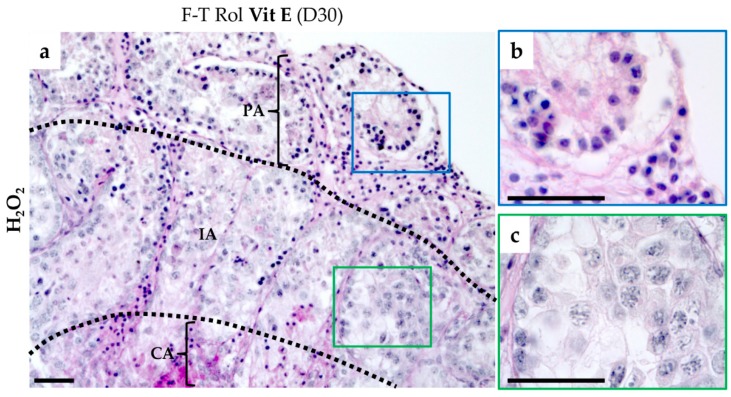
Histological view of the different regions of the cultured testis tissue pre-incubated with H_2_O_2_. (**a**) The peripheral area (PA) showed seminiferous tubules with totally pyknotic nuclei (**b**) due to direct contact with H_2_O_2_. The intermediate area (IA, black dotted lines) showed germ cell differentiation (**c**) with less pyknotic nuclei due to low access of H_2_O_2_ that could explain the low signal intensity for “cytoplasmic” (orange reagent) and “nuclear” (green reagent) ROS generation in this area of the cultured tissue. The central area (CA) showed no germ cell differentiation with pyknotic nuclei due to low oxygen access and coincided with signal fluorescence for ROS detection. Photomicrographs were captured at a ×200 (**a**) or ×1000 (**b**,**c**) magnification. The scale bar represents 40 µm in the photomicrographs. CA, central area; H_2_O_2_, hydrogen peroxide; IA, intermediate area; PA; peripheral area; ROS: reactive oxygen species.

For testicular tissue explants, the peripheral fluorescence intensity due to cytoplasmic and nuclear ROS was markedly increased in the presence of H_2_O_2_, confirming that the signal was due to ROS (Figure 2). In addition, the peripheral area of each testicular fragment contained highly pyknotic nuclei due to a direct contact with H_2_O_2_ (Figure 3). In contrast, the intermediate area showed low signal intensity for ROS detection, coinciding with germ cell differentiation and containing less pyknotic nuclei.

### 2.2. Analysis of ROS Generation in Fresh or F-T Mouse Prepubertal Testicular Tissue during In Vitro Maturation without and with an Antioxidant Supplementation

#### 2.2.1. Evaluation of ROS without Antioxidant Supplementation and Long-Term Culture (60 days)

**Figure 4:**
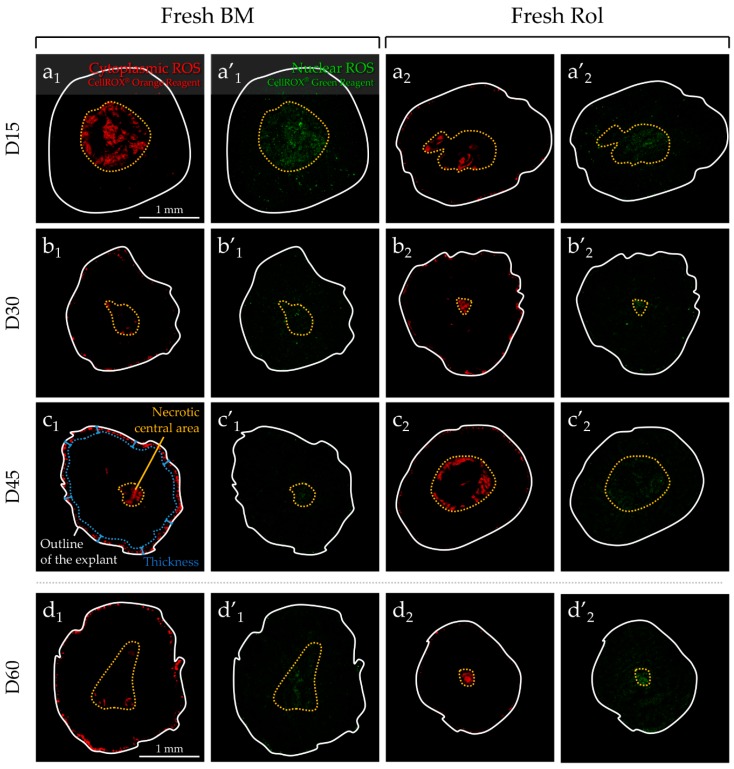

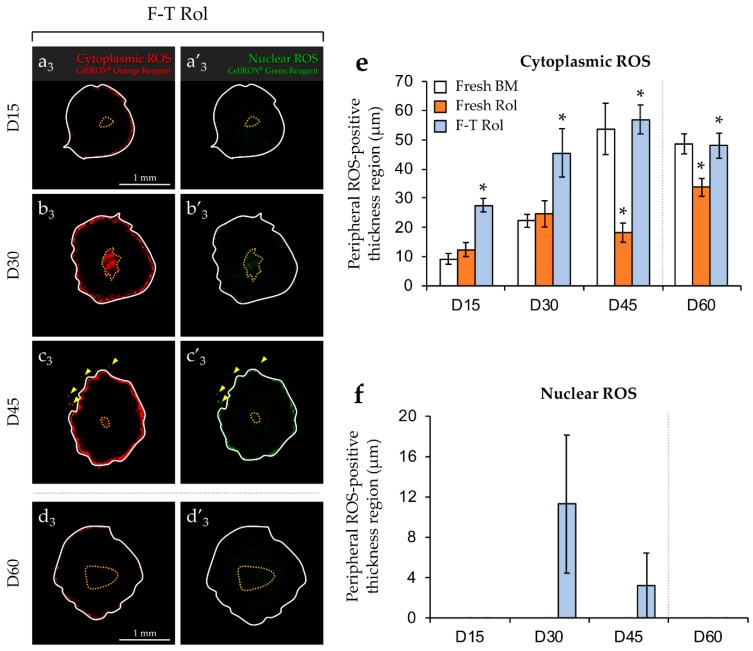
In situ detection of “cytoplasmic” and “nuclear” ROS generation at D15, D30, D45, and D60 in culture of fresh or frozen-thawed prepubertal testicular tissue using ex vivo imaging by confocal macroscopy. Fresh testicular fragments were cultured with (**a_1_**,**a’_1_**,**b_1_**,**b’_1_**,**c_1_**,**c’_1_**,**d_1_**,**d’_1_**) or without (**a_2_**,**a’_2_**,**b_2_**,**b’_2_**,**c_2_**,**c’_2_**,**d_2_**,**d’_2_**) Rol (retinol, vitamin A), whereas F-T testicular fragments were cultured only with Rol (**a_3_,a’_3_,b_3_,b’_3_,c_3_,c’_3_,d_3_,d’_3_**). (**e**) The solid line delineates the outline of the testicular explant. The central necrotic area is delimited with a yellow dotted circle. ROS-positive cells (yellow arrows) were detected outside of the testicular explant at D45 for F-T Rol condition. Decreased fluorescence intensity was obtained for fresh testicular tissue with Rol when compared with BM at D45 and D60. (**f**) Increased fluorescence intensity was observed for F-T testicular fragments with Rol when compared with fresh tissue with Rol at D30 and D45. Green signal fluorescence was observed in a tight peripheral region of the F-T testicular tissues at D30 and D45 of culture. To visualize “cytoplasmic” (orange) and “nuclear” (green) ROS generation in testicular explants, the preparation was illuminated at 532 and 488 nm wavelength, respectively; fluorescence emission was detected from 550 to 580 nm and 500 to 530 nm, respectively. The values (µm) are expressed as the mean of the peripheral ROS-positive thickness region ± standard error of the mean (s.e.m.) of “cytoplasmic” or “nuclear” ROS under the different culture conditions tested, with *n* = 4. Asterisks indicate a statistically significant difference between fresh BM and fresh Rol or between fresh Rol and F-T Rol (* *p* < 0.05). BM, basal medium; D, day; F-T, frozen-thawed; Rol, retinol.

“Cytoplasmic” ROS were detected at the peripheral region of each fresh (Figure 4a_1-2_,b_1-2_,c_1-2_,d_1-2_) or F-T (Figure 4a_3_,b_3_,c_3_,d_3_) testicular tissue fragment, whatever the culture time point. “Nuclear” ROS signal was totally absent in the peripheral region of fresh basal medium (BM) (Figure 4a’_1_,b’_1_,c’_1_,d’_1_) or fresh Rol (Figure 4a’_2_,b’_2_,c’_2_,d’_2_), whatever the culture time point tested, whereas F-T Rol showed a low signal at the periphery at D30 and D45 of culture (Figure 4b’_3_,c’_3_). The peripheral ROS-positive thickness (PRT) region of “cytoplasmic” ROS was significantly higher in fresh BM when compared with fresh Rol at D45 (*p* = 0.01) and D60 (*p* = 0.02) (Figure 4e). However, the highest PRT of “cytoplasmic” ROS was observed in F-T Rol when compared with fresh Rol at D15 (*p* = 0.01), D30 (*p* = 0.01), D45 (*p* = 0.01), and D60 (*p* = 0.02). Moreover, the PRT of “cytoplasmic” ROS increased significantly between D15 and D60 for fresh BM (*p* = 0.0045), fresh Rol (*p* = 0.02), and F-T Rol (*p* = 0.04). Furthermore, some ROS-positive cells were detected outside of the testicular explant for F-T Rol at D45 (Figure 4c_3_,c’_3_). “Nuclear” ROS production (Figure 4f) did not vary significantly between D15 and D60. For the rest of the study, the impact of antioxidant supplementation was assessed for testicular explants cultured until D10, D30, and D45.

#### 2.2.2. Evaluation of Antioxidant Supplementation

**Figure 5:**
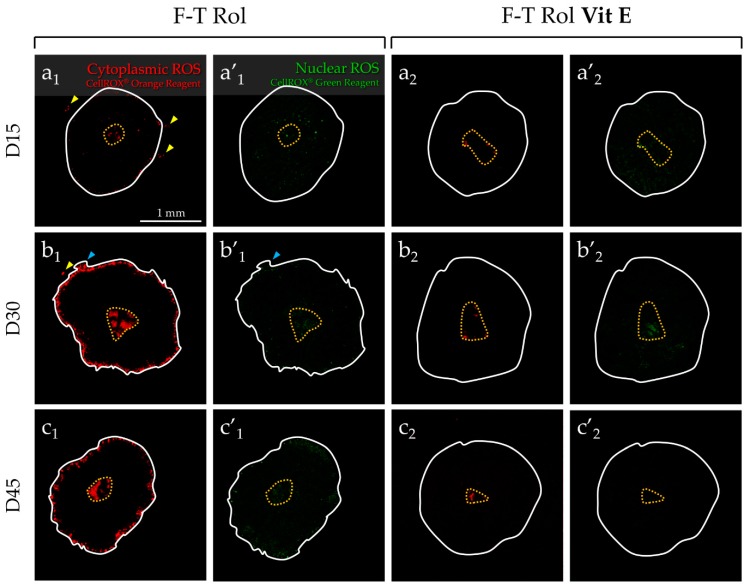

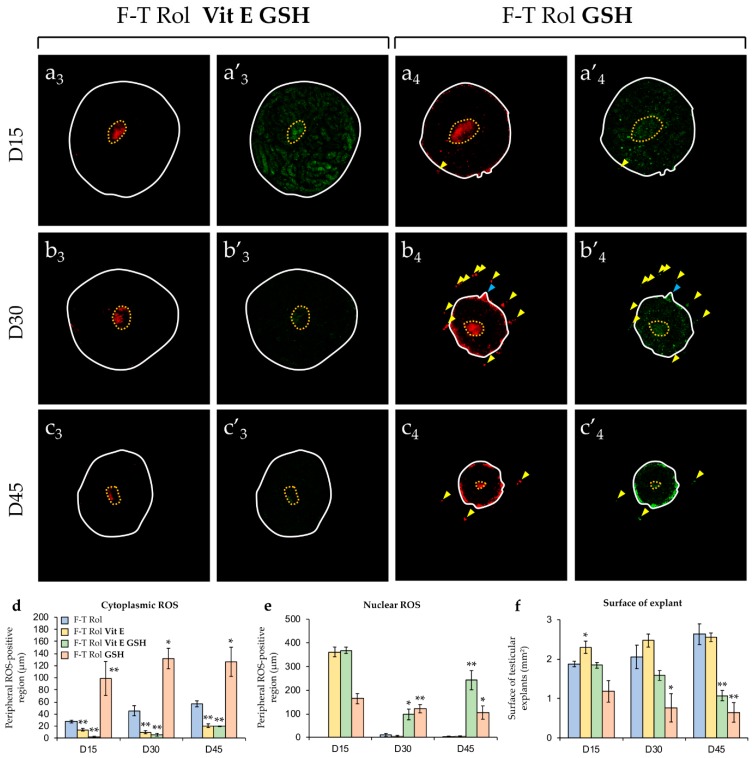
Effect of antioxidant supplementation on “cytoplasmic” and “nuclear” ROS generation in frozen-thawed prepubertal mouse testicular tissue at D15, D30, and D45 of culture using ex vivo imaging by confocal macroscopy. F–T testicular tissues were assessed for “cytoplasmic” ROS generation without (**a_1_**,**a’_1_**,**b_1_**,**b’_1_**,**c_1_**,**c’_1_**) or with (**a_2_**,**a’_2_**,**b_2_**,**b’_2_**,**c_2_**,**c’_2_**) Vit E (vitamin E), Vit E in combination with glutathione (GSH) (**a_3_**,**a’_3_**,**b_3_**,**b’_3_**,**c_3_**,**c’_3_**), or GSH (**a_4_**,**a’_4_**,**b_4_**,**b’_4_**,**c_4_**,**c’_4_**). (**d**) The solid line delineates the outline of the testicular explant. The central necrotic area is delimited with a yellow dotted circle. ROS-positive cells (yellow arrows) with the presence of cells outgrowths (blue arrows) were detected outside of the testicular explant for F–T Rol and F-T Rol GSH conditions. Orange signal (orange-fluorescent redox cytoplasmic dye) was detected in the peripheral region of the testicular explants but also in their necrotic central area that was not taken into consideration for fluorescence measurements and is delimited with a yellow dotted circle. Significant decrease of “cytoplasmic” signal intensity could be observed for F-T Rol Vit E when compared with F-T Rol alone, mainly at D30 and D45 of culture. (**e**) Tight peripheral region showing that orange signal could be observed for F-T Rol Vit E GSH at D15, D30, and D45 of culture. However, high signal intensity could be observed for F-T Rol GSH with reduced surface of the tissues mainly at D30 and D45 of culture. Tight peripheral region showing green signal could be observed for F–T Rol at D30 and D45 of culture. (**f**) Weak signal intensity was observed on the whole surface of testicular tissue for F-T Rol Vit E at D15, but the signal intensity significantly decreased at D30 and D45, mainly in the peripheral region of the tissue. High signal intensity was observed for F-T tissue supplemented with Rol, Vit E, and GSH at D15, although it seemed that the signal decreased at D30 and D45. Green signal was observed for F-T Rol GSH at D15 and D30, but increased significantly at D45. The surface of testicular explants were obtained with the detection of a region of interest by the software. A reduction of the explants cultured with F-T Rol Vit E GSH and F-T Rol GSH could be observed. To visualize “cytoplasmic” (orange) and “nuclear” ROS (green) generation in testicular explants, the preparation was illuminated at 530 and 488 nm wavelength, respectively; fluorescence emission was detected from 550 to 580 nm and 500 to 530 nm, respectively. The values (µm) are expressed as the mean of the peripheral ROS-positive thickness region ± s.e.m. of “cytoplasmic” or “nuclear” ROS under the different culture conditions tested, with *n* = 4. Asterisks indicate a statistically significant difference between fresh BM and fresh Rol or between fresh Rol and F-T Rol (* *p* < 0.05 and ** *p* < 0.01). D, day; F-T, frozen-thawed; GSH, reduced glutathione; Rol, retinol; Vit E, vitamin E.

In contrast of explants cultured with the F-T Rol condition (Figure 5a_1_–c_1_), the “cytoplasmic” ROS signal was weak in the peripheral region of F-T Rol Vit E, regardless of the culture time point (Figure 5a_2_–c_2_). However, the “cytoplasmic” ROS signal was highly detected in F-T Rol Vit E GSH (Figure 5a_3_–c_3_) and in F-T Rol GSH (Figure 5a_4_–c_4_). Moreover, a large population of ROS-positive cells (yellow arrows) with the presence of cell outgrowths (blue arrows) were detected outside of the testicular explants for F-T Rol GSH condition. A faint detection of “nuclear” ROS signal was obtained in testicular fragments with F-T Rol Vit E (Figure 5a’_2_–c’_2_). However, the “nuclear” ROS signal was highly detected on the whole surface of F-T Rol Vit E GSH (Figure 5a’_3_–c’_3_) and F-T Rol GSH (Figure 5a’_3_–c’_3_) when compared with either F-T Rol or F-T Rol Vit E. The PRT of cytoplasmic ROS (Figure 5d) was significantly lower for F-T Rol Vit E when compared with F-T Rol at D15, D30, and D45 (*p* = 0.0048 each). In addition, the PRT of “cytoplasmic” ROS levels was significantly decreased for F-T Rol Vit E GSH when compared with F-T Rol at D15, D30, and D45 (*p* = 0.0048 each). However, the PRT of “cytoplasmic” ROS was significantly increased for F-T Rol GSH when compared with F-T Rol at D15 (*p* = 0.0048), D30 (*p* = 0.019), and D45 (*p* = 0.049). “Cytoplasmic” ROS significantly increased for F-T Rol (*p* = 0.038) and F-T Rol Vit E GSH (*p* = 0.0019) between D15 and D45; however, it did not vary significantly for F-T Rol Vit E (*p* = 0.059) and F-T Rol GSH (*p* = 0.4). Surprisingly, the PRT of “nuclear” ROS (Figure 5e) was higher for F-T Rol Vit E, F-T Rol GSH, and F-T Rol Vit E GSH than for F-T Rol at D15 (*p* < 0.05); however, after a huge decrease for the medium supplemented with Vit E, it was similar between F-T Rol and F-T Rol Vit E at D30 and D45. However, for longer term culture, the PRT of “nuclear” ROS was significantly higher for F-T Rol GSH and F-T Rol Vit E GSH when compared with F-T Rol at D30 (*p* = 0.0048 and *p* = 0.03, respectively) and D45 (*p* = 0.019 and *p* = 0.0048, respectively).

#### 2.2.3. Growth of F-T Testicular Explants during In Vitro Culture with an Antioxidant Supplementation

The mean surface of the F-T testicular explants did not vary significantly with a medium not supplemented with antioxidant throughout time (*p* = 0.23) (Figure 5f), whereas the mean surface significantly increased in F-T Rol Vit E at D15 (*p* = 0.03) but not at D30 (*p* = 0.17) or D45 (*p* = 0.38). However, testicular explants’ area decreased significantly between D0 and D45 for F-T Rol Vit E GSH (*p* = 0.0051) and F-T Rol GSH (*p* = 0.049). In addition, the testicular explants’ area was significantly lower in F-T Rol GSH when compared with F-T Rol, at D30 (*p* = 0.03) and D45 (*p* = 0.0095). Finally, the testicular explants’ area was significantly lower at D45 in F-T Rol Vit E GSH compared with F-T Rol (*p* = 0.0048).

### 2.3. Spermatogenesis Evaluation

#### 2.3.1. Testicular Germ Cells Differentiation

**Figure 6:**
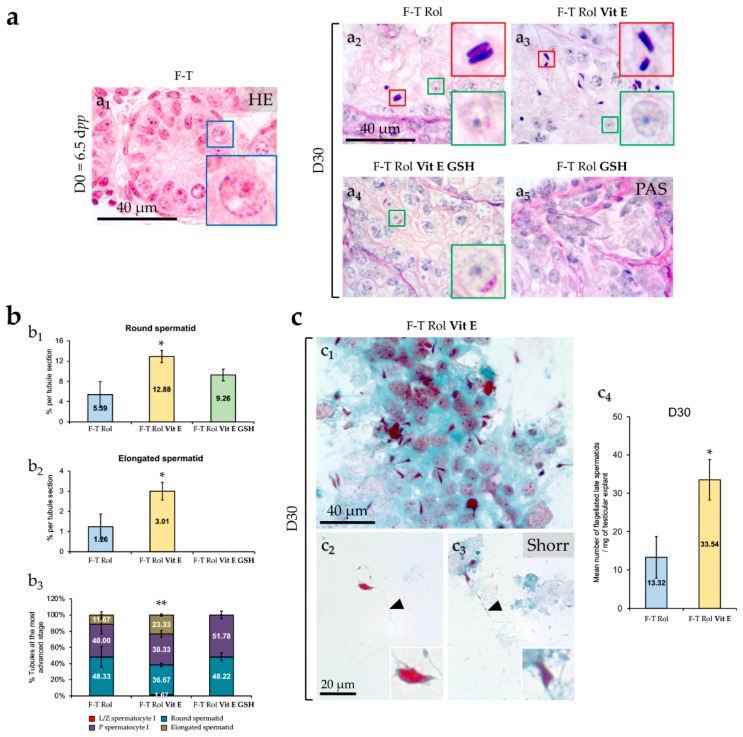
Effect of antioxidant supplementation on in vitro maturation of frozen-thawed prepubertal mouse testicular fragments during the first wave of spermatogenesis. (**a**) Histological evaluation of germ cell differentiation into spermatids according to the culture conditions tested. At D0, seminiferous tubule sections, stained with hematoxylin eosin, contained only spermatogonia (blue boxes) as germ cell (**a_1_**). F-T prepubertal testicular fragments cultured without antioxidant supplementation were used as controls (**a_2_**). At D30 of culture, round spermatids (green boxes) were produced from F-T spermatogonial stem cells (SSCs) and were histologically identified with periodic acid Schiff reaction for F-T Rol (**a_2_**), F-T Rol Vit E (**a_3_**), and F-T Rol Vit E GSH (**a_4_**), but not for F-T Rol GSH, where no germ cell differentiation occurred (**a_5_**). Elongated spermatids (red boxes) were produced in F-T Rol and F-T Rol Vit E. Photomicrographs were captured at a ×1000 magnification (**a_1_**–**a_5_** and insets). (**b**) Stereological analysis of F-T testicular tissue with or without antioxidant supplementation. The mean proportions of round and elongated spermatids according to the conditions tested are shown in (**b_1_**) and (**b_2_**), respectively. The mean proportion of tubules at the most advanced stage according to the conditions tested is shown in (**b_3_**). The values are expressed as the mean percentage ± s.e.m, with *n* = 4 (without antioxidants) or *n* = 6 (with antioxidants) explants. The results were compared between F-T Rol and F-T Rol Vit E, or between F-T Rol and F-T Rol Vit E GSH. No comparison was performed between F-T Rol and F-T Rol GSH, as the latter showed no germ cell differentiation. Asterisks indicate a statistically significant difference (* *p* < 0.05 and ** *p* < 0.01). (**c**) Identification and enumeration of spermatozoa in F-T prepubertal mouse testicular fragments with or without Vit E at D30. After mechanical dissection of the cultured explants, multiple elongated spermatids (**c_1_**) and spermatozoa (**c_2_**,**c_3_**) were evidenced with the use of a culture medium supplemented with Rol and Vit E using Shorr coloration. Enumeration of spermatozoa generated in vitro from F-T prepubertal testicular fragments with or without Vit E is shown in (**c_4_**). Photomicrographs were captured at a ×500 (**c_1_**) and ×1000 (**c_2_**,**c_3_** and insets) magnification. Asterisks indicate a statistically significant difference between F-T Rol and F-T Rol Vit E (* *p* < 0.05). D, day; dpp, day post-partum; F-T, frozen-thawed; GSH, reduced glutathione; HE, hematoxylin eosin; L/Z, leptotene/zygotene; *p*, pachytene; PAS, periodic acid Schiff; Rol, retinol; SSC, spermatogonial stem cell; Vit E, vitamin E.

At D0 of culture, F-T testicular tissues of 6.5 dpp mice contained only spermatogonia (cells with large spherical nuclei at the basement membrane of seminiferous tubules) (Figure 6a_1_). At D30 of culture, germ cell differentiation was observed for all the conditions tested (Figure 6a_2–_a_4_) except for F-T Rol GSH showing a disorganized seminiferous epithelium (Figure 6a_5_). Round spermatids were observed for F-T Rol (Figure 6a_2_), F-T Rol Vit E (Figure 6a_3_), and F-T Rol Vit E GSH (Figure 6a_4_), whereas elongated spermatids were only observed for F-T Rol (Figure 6a_2_) and F-T Rol Vit E (Figure 6a_3_). Stereological analysis showed that the mean percentage of round and elongated spermatids per seminiferous tubule was significantly increased in F-T Rol Vit E when compared with F-T Rol at D30 (*p* = 0.033, both) (Figure 6b_1,_b_2_, respectively). The mean percentage of tubules containing round spermatids was similar between F-T Rol and F-T Rol Vit E, whereas the percentage of tubules containing elongated spermatids was higher for F-T Rol Vit E than for F-T Rol at D30 (*p* = 0.0095) (Figure 6b_3_). However, no significant differences in the mean percentage of round spermatids per tubule section and in the mean percentage of tubules containing round spermatids were found between F–T Rol and F-T Rol Vit E GSH at D30 (Figure 6b_1,_b_3_). Stereological analysis was not performed for F–T Rol GSH, as no germ cell differentiation was observed at D30 (Figure 6a_5_).

#### 2.3.2. Identification and Enumeration of Spermatozoa

Stereological analysis showed that F-T Rol Vit E was the condition that allowed the best in vitro production of round and elongated spermatids from F-T mouse prepubertal testis. Therefore, F-T Rol Vit E was selected for the enumeration of spermatozoa at D30 of culture. F-T Rol Vit E allowed the production of multiple elongated spermatids that remained partially attached to seminiferous epithelium debris at D30 (Figure 6c_1_). Furthermore, F–T Rol Vit E allowed the generation of spermatozoa, which were evidenced by a Shorr coloration at D30 (Figure 6c_2,_c_3_). Mouse spermatozoa with hook-shaped heads were enlarged in the inset of each photomicrograph, whereas the flagellum is shown with a black arrowhead (Figure 6c_2_,c_3_). Sperm enumeration showed that the mean number of spermatozoa was significantly increased with F-T Rol Vit E when compared with F-T Rol at D30 (*p* = 0.02) (Figure 6c_4_).

## 3. Discussion

Several studies reported the relationship existing between freezing and thawing procedures and the high ROS levels in mammalian reproductive cells, mostly in post-thaw spermatozoa [40] and exceptionally in F-T prepubertal testicular tissue [39]. The main findings of the current study were the increased ROS production in F-T prepubertal testicular tissue during in vitro culture when compared with the fresh tissue control. In addition, we demonstrated that Vit E, but not GSH, decreased not solely ROS accumulation in F-T testicular tissue, but also enhanced germ cell differentiation into spermatozoa during the first wave of in vitro spermatogenesis.

The originality of the current study was to use an ex vivo living imaging by confocal macroscopy to explore, in a spatio-temporal manner, the relationship between ROS formation and the ability of F-T SSCs to differentiate in vitro. A major advantage of this technology was to provide appropriate and constant environmental parameters for testicular fragments during observation using a chamber at 34 °C with a constant gas flow (5% CO_2_) and to preserve a homogeneous oxygen distribution within the tissue essential to limit ROS generation. Another advantage of the confocal macroscopy is the large field of view and consequently this technology allows the observation of the entire testicular explants. In addition, we used CellROX probes, which have been reported in several studies for specific detection of cytoplasmic, nuclear, or mitochondrial ROS in a wide range of cells, such as retinal ganglion cells [41,42], macrophage-derived foam cells [43], human lung epithelial cells [44], murine primary cortical neurons [45], Chinese hamster ovary cells [46], or in tissue such as rat Corti organ [47]. These probes have never been used for ROS detection in testicular cells or tissue after thawing or during in vitro culture. Our results are in agreement with those reported in the published data concerning other cell types, as we showed the specific compartmentalization of ROS in testicular cell suspension that allowed us to specifically explore ROS generation in testicular tissue. To reduce the potential phototoxicity of live cell imaging, probes with long wavelength excitation light were used, exposure of the fluorescently labeled cells to illumination was not repeated, and the total excitation light dose was kept to the minimum.

In the current study, we showed that cytoplasmic ROS accumulation was significantly decreased in fresh testicular tissue cultured with Rol when compared with BM alone at D45 and D60 of culture, whereas nuclear ROS were not detected under these two conditions. Rol belongs to the β-carotene family, which is proposed to be a natural antioxidant able to trap and neutralize free radicals and prevent oxidative stress [48]. However, despite Rol supplementation in the culture medium, the highest cytoplasmic ROS accumulation was obtained in F-T tissue between D15 and D60. Therefore, freezing and thawing steps are associated with increased levels of ROS during in vitro culture of F-T testicular tissue. Cryopreservation is known to be extremely damaging to cells and tissues. For example, it has been reported in post-thaw spermatozoa that the freeze–thaw procedure induced ROS formation responsible for changes in membrane function and structure [14,49] and altered the antioxidant defense system [35,50]. In addition, post-thaw SSCs accumulated ROS, had a decreased survival, had a higher rate of apoptosis compared to fresh cells, and had a lower ability in colonizing seminiferous tubules [32,33]. We can suggest that the tissues frozen and thawed using our usual protocols suffered from post-thaw structural damages that allowed higher ROS generation during in vitro culture when compared with the fresh Rol control, whatever the culture time point tested. In addition, three factors could contribute to the in vitro accumulation of ROS: (i) the impairment of endogenous defense mechanisms, (ii) the exposure of cells or tissue to various manipulations, and (iii) an environment that is susceptible to oxidative stress [40]. Therefore, we developed an antioxidant strategy to overcome excessive ROS accumulation in F-T testicular tissue and to improve the in vitro spermatogenesis yield.

The basic cryopreservation, thawing, and culture media used in the current study did not include any Vit E or GSH, except for KnockOut Serum Replacement (KSR) that contained a very low concentration of GSH [51]. Our results demonstrated that Vit E, when added to the three media cited above, had a beneficial effect on ROS generation in F-T testicular tissue because the PRT of cytoplasmic and nuclear ROS was significantly reduced when compared with F-T Rol control, mainly at D30 and D45 of culture. In addition, the structural integrity of F-T testicular tissue was maintained in the presence of Vit E, with a significant increase in the surface of the fragment during in vitro culture between D0 and D45. It is well established that Vit E is a naturally-occurring, liposoluble antioxidant. The α-tocopherol used in the current study is known as the main Vit E active form, and quenches hydrogen peroxide, superoxide anion, and hydroxyl anions, and breaks peroxidation chain reactions [40]. Vit E, when supplemented into the extender, decreased ROS generation and inhibited their deleterious effects on bovine sperm [30]. Similar data were reported in bovine [52] and boar [53,54] spermatozoa with reduced ROS generation when Vit E was added into the extender. We recently showed that, concerning the impact of culture media composition on apoptosis and autophagy repercussions on testicular explant, a basic medium supplemented with Rol and Vit E was selected as the best culture medium for fresh 6.5 dpp tissue cultured during 30 days with 27.7 ± 8.10% of seminiferous tubules containing elongated spermatids [20]. Moreover, adding Vit E to the basic freezing medium increased the quality and viability of isolated SSCs after cryopreservation [33] and reduced ROS production and apoptosis [32]. In addition, the relative protein level expression ratio of an apoptotic factor, the phosphorylated Fas-associated protein with death domain on Fas, was reduced by 64-fold in vitrified testes cultured with BM Rol Vit E [20]. In contrast, we showed in the present study that cytoplasmic and nuclear ROS accumulation were increased in F-T testicular tissue supplemented with GSH. Antioxidant supplementation is not without risk, as adverse events can occur with excess intake of supplements [55]. It has been well established that GSH is able to form disulfide bonds with cysteine residues of proteins, and the relevance of this mechanism (‘‘S-glutathionylation’’) in the regulation of protein function is currently receiving confirmation in a series of research lines. However, rather paradoxically, it has also been highlighted the ability of GSH and notably of its catabolites to promote oxidative processes by participating in metal ion-mediated reactions eventually led to the formation of ROS and free radicals [56]. Our results showed that GSH negatively impacted tissue integrity, as significant disintegration with a major decrease of testicular explant volume was observed during in vitro culture.

Moreover, the effect of antioxidant supplementation on F-T SSC differentiation into spermatozoa was also investigated in the current study. The production of spermatids and spermatozoa from F-T prepubertal testicular tissue was significantly increased with Vit E when compared with the F-T Rol control. It was previously demonstrated that Vit E is a powerful lipophilic antioxidant that is absolutely vital for the maintenance of mammalian spermatogenesis [57]. Vit E is present in particularly high amounts in Sertoli cells and pachytene spermatocytes and to a lesser extent in round spermatids [58]. Vitamin C (Vit C, ascorbic acid), already present in α-minimum essential medium (α-MEM) culture medium, also contributes to the support of spermatogenesis at least in part through its capacity to reduce Vit E and maintain this antioxidant in an active state. Deficiencies of Vit C or E lead to a state of oxidative stress in the testes that disrupts both spermatogenesis and the production of testosterone [57]. In the current study, decreased ROS levels with Vit E coincided with an increased yield of in vitro spermatogenesis when compared with F-T Rol control at D30 of culture. The testis contains large quantities of highly unsaturated fatty acids [11], and Vit E is an efficient antioxidant and the most potent scavenger of lipid peroxyl radicals [59]. Indeed, seminiferous tubules are directly exposed to air in the gas-liquid interphase culture system. Nonetheless, we recently showed that no increased generation of spermatozoa containing 8-OHdG adducts was found in cultures of fresh tissues supplemented with Vit E [60]. Therefore, it can be suggested that the structural integrity of post-thaw testicular cell membranes is better preserved in the presence of Vit E that facilitates inter-communication between the different cell types necessary for the good course of spermatogenesis during in vitro culture of the F-T testicular tissue. However, more spermatozoa presented oxidative damages in cultures of F-T tissues, suggesting that cryopreservation procedures may alter the defensive capacity of undifferentiated and differentiated germ cells against oxidative stress [60].

## 4. Materials and Methods

### 4.1. Mice and Testes Collection

All experiments were approved by the Regional Ethical Committee of Normandy for Animal Care and Use under licence N/23-11-12/46/11-15 on November 2012. CD-1 mice (Charles River Breeding Laboratories, L’Arbresle, France) aged 6.5 days post-partum (dpp) were euthanized by decapitation. The testes were excised and rinsed in Leibovitz L-15 medium (Eurobio, Courtaboeuf, France). After removing the tunica albuginea using sterile needles, the testes were rinsed in the medium. For each litter, one fresh testis was fixed in Bouin’s solution (Sigma-Aldrich, Saint-Quentin Fallavier, France) to ensure with colorations that the seminiferous cords contained no germ cells more advanced than spermatogonia.

### 4.2. Media and Reagents

**Figure 7:**
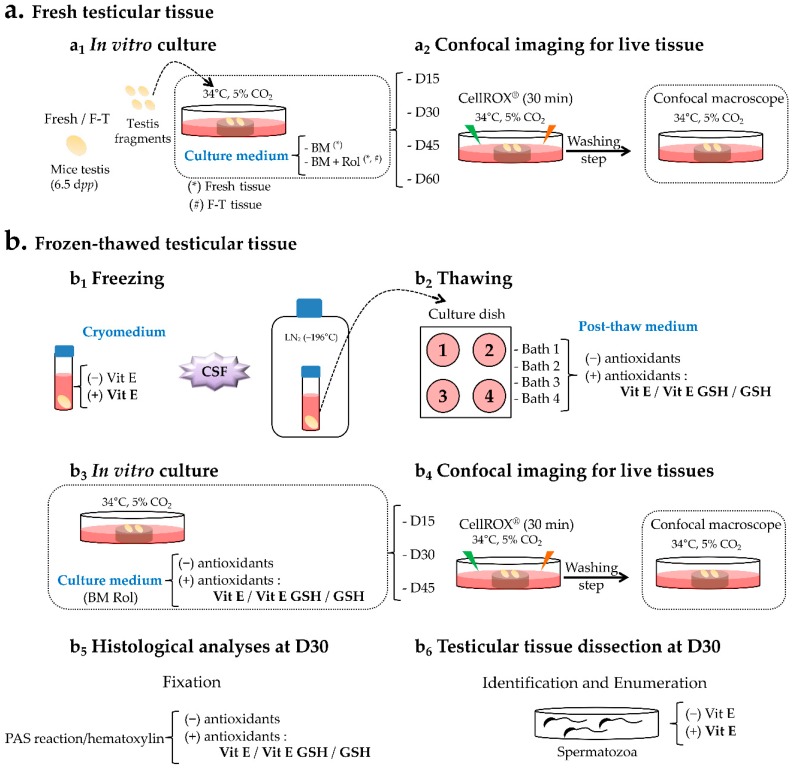
In vitro culture procedure and post-culture analysis using fresh and frozen-thawed prepubertal mouse testes with or without antioxidant supplementation. (**a**) Schematic overview of in vitro culture procedure of fresh and F-T prepubertal mouse testicular tissue (**a_1_**) combined with live confocal imaging for tissue cultured until D15, D30, D45, and D60 of culture (**a_2_**). (**b**) Scheme showing the freezing, thawing, and in vitro culture procedures of prepubertal mouse testis combined with the set of experiments performed with or without antioxidant supplementation. Prepubertal mouse testes were frozen in a cryopreservation medium supplemented or not supplemented with Vit E, using a controlled slow freezing protocol before storage into liquid nitrogen (**b_1_**). Post-thaw medium consisted of four baths containing or not containing Vit E, Vit E combined with GSH, or GSH (**b_2_**). F-T prepubertal testicular fragments were cultured with a medium supplemented or not supplemented with antioxidant(s) (**b_3_**). Confocal live imaging of the cultured tissue was performed at D15, D30, and D45 (**b_4_**). Cytoplasmic and nuclear ROS formation during in vitro culture of testicular fragments was detected using the CellROX kit after incubation during 30 min at 34 °C, 5% CO_2_ (**a_2_,b_4_**). At D30 of culture, histological analysis was performed to quantify the yield of in vitro spermatogenesis using periodic acid Schiff (PAS) reaction counterstained with hematoxylin (**b_5_**). Spermatozoa were identified and counted using Shorr coloration after mechanical dissection of the cultured F-T testicular fragments at D30 in culture medium supplemented or not supplemented with Vit E (**b_6_**). (*), Media tested with fresh explant; (#), media tested with F-T explant; (–), absence; (+), presence; BM, basal medium; CSF, controlled slow freezing; D, day; dpp, day post-partum; F-T, frozen-thawed; GSH, reduced glutathione; LN_2_, liquid nitrogen; min, minutes; PAS, periodic acid Schiff; Rol, retinol; Vit E, vitamin E.

For the fresh testicular tissue, two culture media were compared (Figure 7a) to validate the impact of Rol on cultured pre-pubertal testicular explants: (i) a basal medium (BM) that contained α-minimum essential medium (α-MEM; Life Technologies, Carlsbad, USA), 10% (*v*/*v*) KSR (Life Technologies), and 5 µg/mL gentamicin (Sigma-Aldrich) (Fresh BM) and (ii) a BM supplemented with Rol (Sigma-Aldrich) at a final concentration of 10^−6^ M [18] (Fresh Rol).

For the F-T testicular tissue, four culture media were assessed to evaluate the impact of an antioxidant supplementation on cultured pre-pubertal testicular explants (Table 1): (i) a control culture medium (C) that consisted of BM supplemented with Rol at a final concentration of 10^−6^ M (F-T Rol), (ii) C supplemented with 3.4 mM Vit E (F-T Rol Vit E), (iii) C supplemented with a combination of 3.4 mM Vit E and 5 mM GSH (F-T Rol Vit E GSH), and (iv) C supplemented with 5 mM GSH (F-T Rol GSH).

For the cryopreservation procedure, two freezing media were used (Figure 7b): (i) a basal freezing medium composed of Leibovitz L-15, 1.5 M DMSO (Sigma-Aldrich), 0.05 M sucrose (Sigma-Aldrich), and 10% (*v*/*v*) fetal calf serum (Eurobio); and (ii) a basal freezing medium supplemented with 3.4 mM Vit E (Sigma-Aldrich) [30]. The thawing procedure was performed using four successive baths supplemented or not supplemented with 3.4 mM Vit E or 5 mM GSH (Sigma-Aldrich) [34].

### 4.3. Cryopreservation Procedure

Prepubertal testes were cryopreserved using a controlled slow freezing (CSF) protocol, as previously described [24,61,62]. Briefly, the CSF protocol was performed by placing one testis into a cryovial (Cryotube Vials, Nunc, Roskilde, Denmark) containing 1.3 mL of the freezing medium described above in the absence or presence of Vit E. Equilibration was performed at 4 °C for 30 min. Using a programmable freezer (Nicool Freezal, Air Liquide, Marne La Vallée, France), the vials were cooled using a cooling rate of −2 °C/min from 5 °C until reaching the soaking temperature of −9 °C, stabilized for 8 min, followed by a rate of −0.3 °C/min to −40 °C, and finally −25 °C/min to −150 °C [61,62]. Subsequently, the samples were directly plunged and stored into liquid nitrogen at −196 °C for one month.

Frozen testicular tissue samples were thawed rapidly in a 37 °C water bath and placed into several baths with a progressive dilution of cryoprotectant to avoid osmotic stress. Thereafter, the cryomedium was removed in a four-step procedure with 5 min for each step: (i) 1 M DMSO, 0.05 M sucrose, 10% fetal calf serum (FCS); (ii) 0.5 M DMSO, 0.05 M sucrose, 10% FCS; (iii) 0.05 M sucrose; and (iv) Leibovitz medium without cryoprotectant. These washing steps were performed with or without anti-oxidants as described above (see Section 4.2).

### 4.4. Organ Culture

Fresh or F-T testicular tissue were partially inserted in 1.5% (*w*/*v*) agarose gels (Sigma-Aldrich) to prevent slippage. Agarose gel strands were placed into a small Petri dish (Falcon Tissue Culture Dish; BD Biosciences, Franklin Lakes, New Jersey, USA) and pre-soaked overnight in BM to replace water. Fresh testicular tissue was cultured using the BM medium alone (fresh BM) or supplemented with Rol (fresh Rol), whereas F-T testicular tissue was cultured with the “C” medium alone, supplemented or not supplemented with antioxidants as described above (see Section 4.2). The culture medium was changed every two days and replaced by extemporaneously prepared medium including the same components as mentioned in Section 4.2 for each culture conditions. The incubator used for culture contained 5% CO_2_ and was maintained at 34 °C. Four fresh testes from mice of different litters were used for each culture condition tested, and were cultured for 15 (D15), 30 (D30), 45 (D45), and 60 (D60) days. Six F-T testes from mice of different litters were used for each culture condition tested and were cultured until D15, D30, and D45.

### 4.5. Confocal Live Imaging

#### 4.5.1. ROS Measurement in Germinal Cells

ROS were detected using the cell-permeable CellROX orange reagent and CellROX green reagent (Life Technologies, Molecular Probes, Eugene, OR, USA). The CellROX probes are peroxide-sensitive, vital, and fluorogenic probes widely used in toxicology assays. The cell-permeable reagents are non-fluorescent in a reduced state and exhibit strong fluorogenic signal upon oxidation. The fluorogenic, ROS-sensitive dye CellROX green reagent (C10444, Life Technologies) was used to measure superoxide (O2-) and hydroxyl radical (•OH) in live cells, and the dye becomes fluorescent upon binding to DNA after being oxidized by O2^−^ and/or •OH with a signal localized primarily in the nucleus and mitochondria. In contrast, CellROX orange reagent (C10443, Life Technologies) detects cytoplasmic free radicals and consequently the fluorescent signal is localized in the cytoplasm. To verify the specific localization of ROS signal, testicular cell suspension obtained after mechanical dissection of 6.5 dpp mice was cultured overnight at 34 °C in 35 mm glass-bottom microwell dishes (MatTek corporation, Ashland, MA, USA) using BM containing the green/orange CellROX reagents and Hoechst 33342. The fluorescent signal was analyzed with an inverted confocal microscope (TCS SP2 AOBS, Leica) by using an oil immersion ×63 objective (NA = 1.4) and a 405, 488, or 561 nm excitation wavelength for the Hoechst 33342, green, and orange probes, respectively. The fluorescence emission was sequentially detected by setting spectral windows from 460–490 nm for the Hoechst 33342, from 500–530 nm for the green probe, and from 550–580 nm for the orange probe. The temperature of the chamber was kept at 34 °C and cells were provided with constant gas flow (5% CO_2_) during image capture.

#### 4.5.2. ROS Measurement in Testicular Tissue Explants

Culture dishes containing live testicular tissue were incubated with CellROX reagents that were added simultaneously in the culture media cited above at a final concentration of 5 µM each, for 30 min at 34 °C. Thereafter, the medium was removed and replaced by a novel culture considered as a washing step solution. The Petri dish was then directly transferred into an incubator attached to the stand of a confocal macroscope (TCS LSI, Leica Microsystems, Nanterre, France). During the procedure, the temperature of the chamber was kept at 34 °C using a temperature controller (Tempcontrol 37-2 digital 2-channel, PeCon, Ulm, Germany), and the tissues were supplied with constant gas flow (5% CO_2_; CO_2_-controller, PeCon, Ulm, Germany). To visualize ROS generation in testicular tissue, the preparation was illuminated with a 488 (green) or 532 nm (orange) wavelength light by means of a laser diode through a confocal laser scanning macroscope equipped with a ×2 dry objective (NA = 0.22, working distance: 39 mm, diameter: 58 mm, Leica Microsystems). In a sequential mode, the green fluorescence emission was detected from 500–550 nm and the orange fluorescence emission from 540–600 nm. To verify the specificity of the probes for the detection of ROS, testicular tissue was incubated with H_2_O_2_ at a final concentration of 200 µM overnight at 34 °C and was used as a positive control.

The fluorescence signal obtained with each excited probe was principally localized at the peripheral and central regions of the testicular fragment. It is worth noting that the peripheral region of the cultured sample is the preferential site of germ cell differentiation, whereas the central region is completely necrotic due to hypoxia, as previously reported [18]. Then, the peripheral fluorescence signal was assessed by measuring the thickness (in µm) of this peripheral ROS-positive region at eight points for each testis fragment according to the conditions and the time points of culture using software image analysis (LAS AF Lite, Leica).

### 4.6. Histological Analysis

Fresh testes tissue, F-T prepubertal testes, and testicular tissue explants were fixed in Bouin’s solution (Sigma-Aldrich) for 2 h at room temperature, dehydrated in a graded series of ethanol washes, and embedded in paraffin. Sections (3 µm thick) were cut using a microtome (JungRM 2035; Leica Microsystems GmbH, Wetzlar, Germany). For both the 6.5 dpp fresh and F-T testes, slides were stained with hematoxylin eosin (HE) to have an accurate appreciation of seminiferous tubule architecture, as previously described. A periodic acid Schiff (PAS)-hemalun (RAL diagnostic, Martillac, France) reaction was chosen for analysis of the 36.5 dpp in vivo testes and D30 in vitro testicular tissue fragments to detect the characteristic pink-labelled acrosome of round spermatids with regular small round blue nuclei. In addition, elongated spermatids were detected as cells with elongated blue nuclei with highly condensed chromatin. Serial digital images were obtained with a light microscope (DM4000B; Leica Microsystem GmbH) equipped with Leica Application Suite software (Leica Microsystem GmbH). Subsequently, the mean proportion of round and elongated spermatids per tubule section, but also the mean percentage of tubule sections at the most advanced stage of spermatogenesis, were determined in 30-sectioned seminiferous tubules.

### 4.7. Spermatozoa Enumeration

Four F-T testicular explants obtained from 6.5 dpp mice from different litters were cultured for 30 days in culture medium supplemented or not supplemented with Vit E. At the end of the culture period, each testicular explant was weighed, placed into a Petri dish (BD Biosciences) that contained 500 µL of α-MEM at 34 °C with 5% CO_2_, and shredded with sterile needles. Subsequently, 20 µL of each testicular cell suspension was observed under a light microscope at ×400 magnification (Laborlux, Leica) to identify and count spermatozoa. Thereafter, the remaining testicular cell suspension was centrifuged for 10 min at 600 g and the pellet was subjected to Shorr staining (Merck, Darmstadt, Germany).

### 4.8. Statistical Analysis

Statistical analysis was performed for all experiments using the Kruskal–Wallis test for global comparisons and the Mann–Whitney test for unpaired rank comparisons. The data were presented as the mean ± s.e.m.; and a *p*-value below 0.05 was considered statistically significant.

## 5. Conclusions

In conclusion, by using CellROX staining in live testicular tissue, we present evidence that the freeze–thaw procedure, as well as the in vitro culture conditions, increased the production of cytoplasmic ROS within testicular cells. Our results strongly suggest that the prevention of oxidative stress in the cytoplasmic compartment should be regarded as a potential strategy for improving SSC viability and functionality during the freeze–thaw procedure and in vitro maturation. Indeed, Vit E, but not GSH, reduced the generation of ROS after thawing and during in vitro culture. Our slow freezing protocol associated with the antioxidant strategy developed in the current study should be proposed for a clinical application.

## Figures and Tables

**Table 1:** Description of the conditions tested according to the in vitro culture procedures of fresh and frozen-thawed prepubertal mouse testicular tissue without or with antioxidant supplementation. (–), absence; (+), presence; BM, basal medium; F-T, frozen-thawed; GSH, reduced glutathione; Rol, retinol; Vit E, vitamin E.

Conditions	Testicular Tissue	Cryomedium	Post-Thaw Medium	Culture Medium
Fresh BM	Fresh	-	-	BM
Fresh Rol	Fresh	-	-	BM + Rol
F-T Rol	Frozen-thawed	No antioxidant	No antioxidant	BM + Rol
F-T Rol Vit E	Frozen-thawed	With Vit E	With Vit E	BM + Rol + Vit E
F-T Rol Vit E GSH	Frozen-thawed	With Vit E	With Vit E and GSH	BM + Rol + Vit E+GSH
F-T Rol GSH	Frozen-thawed	With GSH	With GSH	BM + Rol + GSH

## Abbreviations

BM	basal medium
CA	central area
CSF	controlled slow freezing
D	day
d*pp*	days post-partum
FCS	fetal calf serum
F-T	frozen-thawed
GSH	reduced glutathione
h	hour
HE	hematoxylin eosin
H_2_O_2_	hydrogen peroxide
IA	intermediate area
KSR	KnockOut Serum Replacement
LN_2_	liquid nitrogen
L/Z	leptotene/zygotene
min	minutes
P	pachytene
PA	peripheral area
PAS	periodic acid Schiff
Rol	retinol
ROS	reactive oxygen species
s.e.m.	standard error of the mean
SSC	spermatogonial stem cell
Vit E	vitamin E
